# Electronic Health Record Documentation of Psychiatric Assessments in Massachusetts Emergency Department and Outpatient Settings During the Coronavirus Disease 2019 (COVID-19) Pandemic

**DOI:** 10.1001/jamanetworkopen.2020.11346

**Published:** 2020-06-08

**Authors:** Victor M. Castro, Roy H. Perlis

**Affiliations:** 1 Center for Quantitative Health, Division of Clinical Research, Massachusetts General Hospital, Boston; 2 Associate Editor, *JAMA Network Open*

## Abstract

This cohort study investigates the documentation of psychiatric symptoms in narrative clinical notes as coronavirus disease 2019 (COVID-19) prevalence increased in eastern Massachusetts.

## Introduction

The emergence of the worldwide coronavirus disease 2019 (COVID-19) pandemic has been associated with increased burden of psychiatric symptoms among health care workers and the general public,^1,2^ which may increase the risk of longer-term sequelae.^3,4^ At the same time, the need for quarantine and the strain on clinical resources may reduce the ability of health systems to respond to such symptoms. To quantify shifts in psychiatric evaluation associated with COVID-19, we used electronic health records from 2 large academic medical centers and 3 affiliated community hospitals in Massachusetts to investigate the documentation of psychiatric symptoms in narrative clinical notes as COVID-19 activity increased in eastern Massachusetts.

## Methods

This cohort study included all individuals seen in outpatient or emergency department visits between January 2 and March 25, 2020. Sociodemographic data, including age, sex, race, and ethnicity, were drawn from the Partners Research Patient Data Registry^5^ along with narrative clinical notes. Presence of depression, anxiety, suicide, psychosis, and violence was determined by identifying terms drawn from an expert-curated list associated with National Institute of Mental Health Research Domain Criteria.^6^ These terms included *depressed*, *depressive*, *dysphoric*, *dysthymic*, *sad*, and *tearful *for depression; *anxiety*, *anxious*, *fearful*, *frighten*, *hypervigilant*, *nervous*, *panic*, *phobia*, *phobic*, *scared*, *stress*, *tense*, and *worried *for anxiety; *suicide*, *suicidal*, and *suicidality *for suicide; *psychotic*, *psychosis*, *hallucination*, *delusion*, *paranoid*, *paranoia*, *hallucinate*, *hallucinated*, and *delusional *for psychosis; and *violence* and *violent *for violence.

Presence of a coronavirus test was determined from the enterprise laboratory feed. The Partners HealthCare human research committee approved the study protocol. Given that no participant contact was required in this study based on secondary use of data arising from routine clinical care, the committee waived the requirement for informed consent as detailed by 45 CFR 46.116. This study followed the Strengthening the Reporting of Observational Studies in Epidemiology (STROBE) reporting guideline.

Primary analysis quantified the volume of documentation by individual symptoms for each week in the study period. Logistic regression was used to examine the association between the presence of COVID-19 testing and psychiatric symptoms, adjusted for age, sex, and race/ethnicity, using R version 3.6.0 (R Project for Statistical Computing). Notes were treated as clustered within individuals and limited to those occurring at or before time of testing. No significance testing was performed.

## Results

A total of 205 957 emergency department and 2 483 159 outpatient notes were analyzed (Table), representing 60 428 patients (32 550 [53.9%] women; mean [SD] age, 44.3 [24.5] years) and 541 307 patients (324 823 [60.0%] women; mean [SD] age, 50.9 [21.7] years), respectively. The Figure illustrates the frequency of COVID-19 testing by week (Figure, A) and the frequency of mentions of psychiatric terms in notes by week (Figure, B and C). The initial 2 decrements correspond to 4-day weeks because of holidays. In January and February, with holidays excluded, there were a mean (SD) of 1446 (79) and 49 312 (4841) notes with mentions of depression from emergency department and outpatient visits, respectively, per week. These decreased to 886 (ie, by 44%) and to 9315 (ie, by 81%), respectively, in the week spanning March 19 to March 25. Similar patterns were observed for other symptoms.

**Table.** Sociodemographic Characteristics of Patients Seen From January 2 to March 25, 2020

Characteristic	No. (%)		
	Emergency department notes (n = 205 957)	Outpatient notes (n = 2 483 159)	Total notes (N = 2 689 116)
Sex			
Women	111 679 (54.2)	1 525 338 (61.4)	1 637 017 (60.9)
Age, mean (SD), y	47.1 (23.5)	52.0 (21.4)	51.7 (21.7)
Race			
Asian	8119 (3.9)	108 327 (4.4)	116 446 (4.3)
Black	30914 (15.0)	164 712 (6.6)	195 626 (7.3)
Other	22 224 (10.8)	133 952 (5.4)	156 176 (5.8)
Unknown	16 128 (7.8)	170 988 (6.9)	187 116 (7.0)
White	128 572 (62.4)	1 905 180 (76.7)	2 033 752 (75.6)
Ethnicity			
Hispanic	10 344 (5.0)	88 524 (3.6)	98 868 (3.7)
Hospital type			
Academic medical center	124 518 (60.5)	1 920 205 (77.3)	2044 723 (76.0)
Community hospital	81 439 (39.5)	562 954 (22.7)	644 393 (24.0)
COVID-19 laboratory order	5635 (2.7)	34 165 (1.4)	39 800 (1.5)
Anxiety term in note	39 978 (19.4)	978 308 (39.4)	1 018 286 (37.9)
Depression term in note	16 810 (8.2)	491 612 (19.8)	508 422 (18.9)
Psychosis term in note	10 272 (5.0)	121 612 (4.9)	131 884 (4.9)
Suicide term in note	11 007 (5.3)	133 022 (5.4)	144 029 (5.4)
Violence term in note	4722 (2.3)	234 381 (9.4)	239 103 (8.9)

Abbreviation: COVID-19, coronavirus disease 2019.

**Figure.**
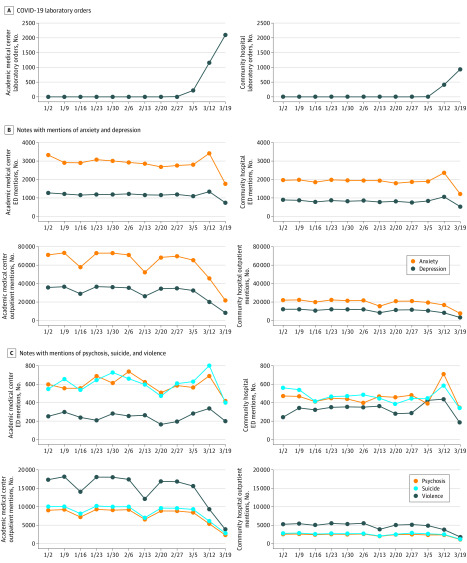
Trends in Coronavirus Disease 2019 (COVID-19) Testing Laboratory Orders and Mentions of Psychiatric Terms from January 2 to March 25, 2020 ED indicates emergency department.

In the emergency department setting, the odds of COVID-19 testing, adjusted for age, sex, race/ethnicity, and hospital type, were increased by nearly 50% in visits during which violence was referenced (odds ratio, 1.49; 95% CI, 1.25-1.76). In the outpatient setting, notes with the presence of psychiatric terms were associated with a reduction in the likelihood of COVID-19 testing for an individual patient, with adjusted odds ratios of 0.80 (95% CI, 0.77-0.82) for anxiety, 0.64 (95% CI, 0.61-0.67) for depression, 0.63 (95% CI, 0.56-0.71) for psychosis, 0.73 (95% CI, 0.66-0.81) for suicide, and 0.40 (95% CI, 0.37-0.43) for violence.

## Discussion

In this cohort study, we detected a marked reduction in notes documenting psychiatric symptoms, inclusive of telemedicine, paralleling the emergence of COVID-19 diagnoses in the Boston area. During this period, outpatient visits were cancelled, individuals likely became more reluctant to come to the emergency department, and clinicians seeking to reduce exposure may have conducted more focused interviews. In emergency department evaluations, most symptoms were not associated with COVID-19 testing, but reference to violence was associated with a greater likelihood of testing, perhaps reflecting a shift in clinical presentations. Limitations of these results include the reliance on simple string matching and the inability to determine generalizability from these 5 hospitals to other regions.

Nonetheless, our results indicate an acute reduction in the assessment and documentation of psychiatric symptoms. In light of survey data,^2^ we might expect to see an increase rather than a decrease in psychiatric presentations in Boston-area hospitals. Efforts to provide more accessible psychiatric care during the acute phase of the COVID-19 pandemic may become particularly important; symptoms are likely to be increasing while access is decreasing. Strategies such as telemedicine are urgently needed to ensure that another consequence of the pandemic is not neglect of psychiatric illness.
